# Functional Metabolic Mapping Reveals Highly Active Branched-Chain Amino Acid Metabolism in Human Astrocytes, Which Is Impaired in iPSC-Derived Astrocytes in Alzheimer's Disease

**DOI:** 10.3389/fnagi.2021.736580

**Published:** 2021-09-17

**Authors:** Claudia Salcedo, Jens V. Andersen, Kasper Tore Vinten, Lars H. Pinborg, Helle S. Waagepetersen, Kristine K. Freude, Blanca I. Aldana

**Affiliations:** ^1^Department of Drug Design and Pharmacology, Faculty of Health and Medical Sciences, University of Copenhagen, Copenhagen, Denmark; ^2^Epilepsy Clinic and Neurobiology Research Unit, Copenhagen University Hospital, University of Copenhagen, Copenhagen, Denmark; ^3^Department of Veterinary and Animal Sciences, Faculty of Health and Medical Sciences, University of Copenhagen, Frederiksberg, Denmark

**Keywords:** AD, astrocytes, BCAA, glutamate, glutamine, neuron, induced pluripotent stem cell, energy metabolism

## Abstract

The branched-chain amino acids (BCAAs) leucine, isoleucine, and valine are important nitrogen donors for synthesis of glutamate, the main excitatory neurotransmitter in the brain. The glutamate carbon skeleton originates from the tricarboxylic acid (TCA) cycle intermediate α-ketoglutarate, while the amino group is derived from nitrogen donors such as the BCAAs. Disturbances in neurotransmitter homeostasis, mainly of glutamate, are strongly implicated in the pathophysiology of Alzheimer's disease (AD). The divergent BCAA metabolism in different cell types of the human brain is poorly understood, and so is the involvement of astrocytic and neuronal BCAA metabolism in AD. The goal of this study is to provide the first functional characterization of BCAA metabolism in human brain tissue and to investigate BCAA metabolism in AD pathophysiology using astrocytes and neurons derived from human-induced pluripotent stem cells (hiPSCs). Mapping of BCAA metabolism was performed using mass spectrometry and enriched [^15^N] and [^13^C] isotopes of leucine, isoleucine, and valine in acutely isolated slices of surgically resected cerebral cortical tissue from human brain and in hiPSC-derived brain cells carrying mutations in either amyloid precursor protein (APP) or presenilin-1 (PSEN-1). We revealed that both human astrocytes of acutely isolated cerebral cortical slices and hiPSC-derived astrocytes were capable of oxidatively metabolizing the carbon skeleton of BCAAs, particularly to support glutamine synthesis. Interestingly, hiPSC-derived astrocytes with APP and PSEN-1 mutations exhibited decreased amino acid synthesis of glutamate, glutamine, and aspartate derived from leucine metabolism. These results clearly demonstrate that there is an active BCAA metabolism in human astrocytes, and that leucine metabolism is selectively impaired in astrocytes derived from the hiPSC models of AD. This impairment in astrocytic BCAA metabolism may contribute to neurotransmitter and energetic imbalances in the AD brain.

## Introduction

**Figure 1:**
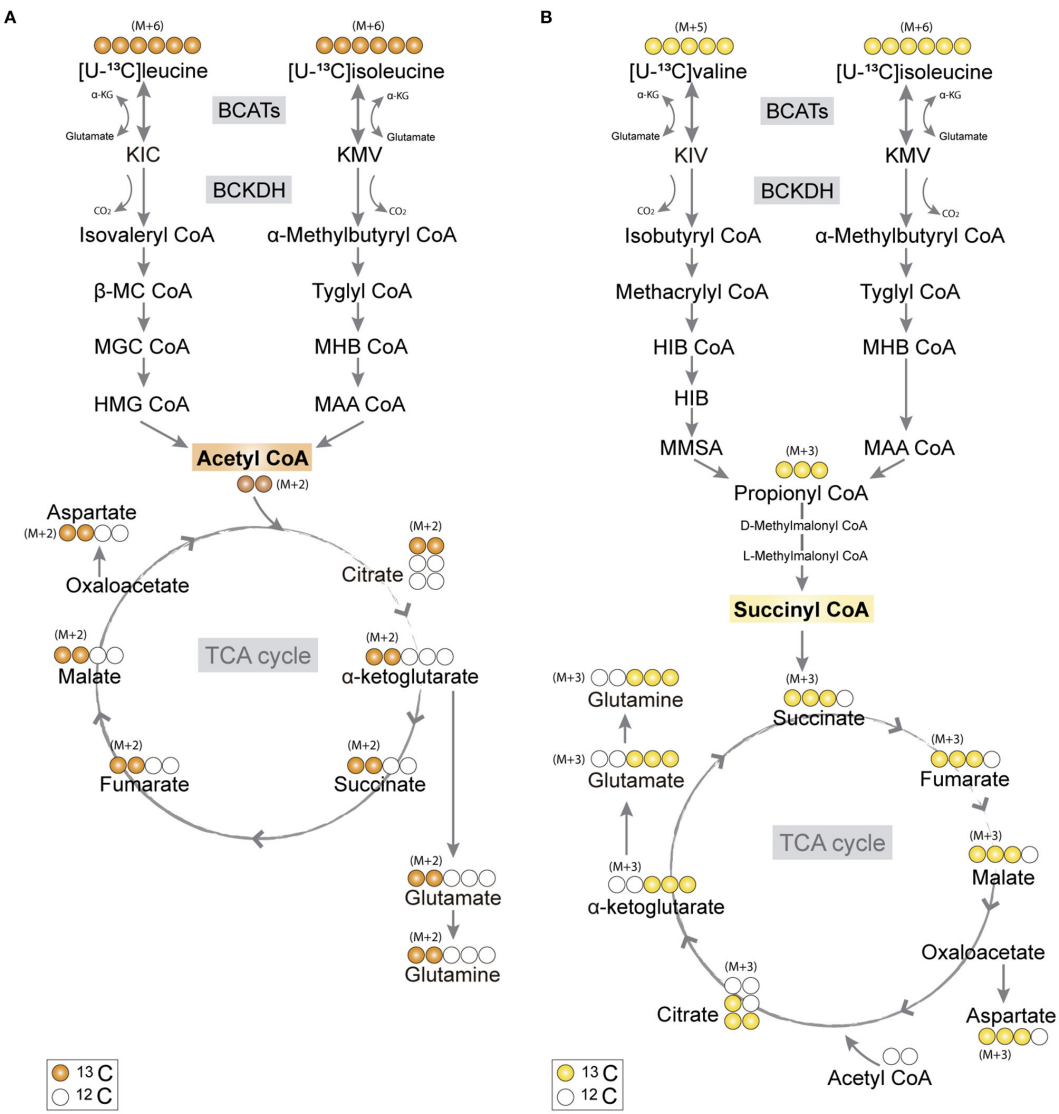
Primary ^13^C-labeling patterns from [U-^13^C]branched-chain amino acid (BCAA) metabolism. **(A)** Metabolism of [U-^13^C]leucine and [U-^13^C]isoleucine (M+6) will result in acetyl coenzyme A (CoA), a process initiated by branched-chain aminotransferase (BCAT) catalyzing BCAA transamination and hereby producing branched-chain α-keto acids (BCKAs), α-ketoisocaproate (KIC), and α-keto-β-methylvalerate (KMV), respectively. Subsequently, irreversible oxidative decarboxylation of the BCKAs occurs by the branched-chain α-keto acid dehydrogenase (BCKDH) enzyme complex and through multiple enzymatic reactions entering the TCA cycle as double-labeled acetyl CoA (M+2). Filled circles (orange) represent labeled ^13^C and empty circles unlabeled ^12^C. **(B)** Valine and isoleucine can also enter into the TCA cycle *via* succinyl CoA. [U-^13^C]valine (M+5) and [U-^13^C]isoleucine (M+6) will be transaminated, resulting in BCKAs, α-ketoisovaleric (KIV), and KMV, respectively, and then decarboxylated, entering TCA cycle metabolism, after numerous reactions, as succinate (M+3). Filled circles (yellow) represent labeled ^13^C and empty circles unlabeled ^12^C. BCATs, branched-chain aminotransferase; BCKDH, branched-chain α-keto acid dehydrogenase; α-KG, α-ketoglutarate; KIC, α-ketoisocaproate; KMV, α-keto-β-methylvalerate; KIV, α-ketoisovaleric; β-MC CoA, β-methylcrotonyl CoA; MGC CoA, β-methylglutaconyl CoA; HIB CoA, β-hydroxyisobutyryl CoA; MHB CoA, α-methyl-β-hydroxyisobutyryl CoA; HMG CoA, β-hydroxy-β-methylglutaryl CoA; MAA CoA, α-methylacetoacetyl CoA; HIB, β-hydroxyisobutyrate; MMSA, methylmalonate semialdehyde; MAA CoA, α-methylacetoacetyl CoA.

Branched-chain amino acids (BCAAs) comprise three essential amino acids, leucine, isoleucine, and valine, which all have multiple functions in the brain (Yudkoff, 1997; Conway and Hutson, 2016). BCAAs are important nitrogen donors essential for nitrogen homeostasis and neurotransmitter cycling (Yudkoff, 1997; Sperringer et al., 2017). Furthermore, BCAAs can be utilized by brain cells as energy substrates in the tricarboxylic acid (TCA) cycle. BCAA metabolism is initiated by reversible transamination catalyzed by the branched-chain amino acid transaminase (BCAT) producing the corresponding branched-chain α-keto acids (BCKAs) and glutamate. BCAT exists as two isozymes, one cytosolic (BCATc/BCAT1), which is only found in brain, ovaries and testes, and one mitochondrial (BCATm/BCAT2), which is expressed in most tissues (Conway and Hutson, 2016). The subsequent irreversible oxidative decarboxylation of BCKAs is catalyzed by the branched-chain α-keto acid dehydrogenase (BCKDH) complex, followed by multiple other reactions (Figure 1), ultimately yielding acetyl coenzyme A (CoA) or succinyl CoA (Sperringer et al., 2017), which may support the TCA cycle and amino acid synthesis. Discrepancies on the cellular location of the metabolic machinery of BCAA metabolism have prompted the suggestion that astrocytes have a limited BCAA metabolism capacity (Conway and Hutson, 2016; Sperringer et al., 2017). Based on immunohistochemical studies, it has been suggested that human astrocytes do not express BCAT or BCKDH (Hull et al., 2012, 2015, 2018), which has led to the conclusion that human astrocytes are incapable of metabolizing BCAAs (Sperringer et al., 2017). However, this matter has not been functionally investigated.

Alzheimer's disease (AD) is a complex neurodegenerative disorder characterized by a heterogeneous pathology comprising defective protein clearance (Braak and Braak, 1991), disrupted energy metabolism (Cunnane et al., 2020), neuroinflammation (Leng and Edison, 2021), and oxidative stress (Butterfield and Halliwell, 2019). AD pathology gradually leads to common clinical manifestations, such as cognitive and memory impairment (Winblad et al., 2016). Although research has mainly focused on neurons, increasing evidence points toward the involvement of non-neuronal cells, namely, astrocytes, microglia, and oligodendrocytes, in the progression of the disease (De Strooper and Karran, 2016; Acosta et al., 2017). However, the glial contribution in AD is yet to be fully unraveled (De Strooper and Karran, 2016; Aldana, 2019; Bogetofte et al., 2019). Alterations in brain glucose metabolism is one of the earliest biomarkers of AD development (Gordon et al., 2018). Brain energy metabolism is closely related to neurotransmission (Yu et al., 2018). Interestingly, disrupted excitatory glutamate signaling is strongly implicated in AD as well as several other neurodegenerative disorders (Liu et al., 2019; Conway, 2020). Only a small fraction of glutamate from the circulating peripheral blood plasma crosses the blood-brain barrier (BBB), thus the majority of brain glutamate must be *de novo* synthesized (Sperringer et al., 2017). In the brain, glutamate is synthesized from TCA cycle intermediate α-ketoglutarate, providing the carbon backbone (Brekke et al., 2016), while the amino group is derived from a nitrogen donor such as BCAAs, aspartate, or alanine (Conway and Hutson, 2016). Particularly, BCAAs play an essential role as nitrogen donors, and they account for approximately one-third of the amino groups used for brain glutamate synthesis (Yudkoff, 1997). Furthermore, the carbon skeleton of BCAAs, entering cellular metabolism as either acetyl CoA or succinyl CoA, may serve as auxiliary fuels for brain cells, hereby supporting the failing energy metabolism in AD (Cunnane et al., 2020). Astrocytes take up the majority of synaptic glutamate released from neurons and hereby play a crucial role in terminating excitatory signaling. In astrocytes, most of the synaptic glutamate is converted into glutamine by the action of the enzyme glutamine synthetase (GS), which is selectively expressed in astrocytes (Norenberg and Martinez-Hernandez, 1979; Schousboe et al., 2013). The glutamine is subsequently released and taken up by neurons, which is essential to replenish the neuronal glutamate pool. This exchange of metabolites between neurons and astrocytes is known as the glutamate-glutamine cycle and is particularly important during extensive glutamatergic signaling (Shen, 2013; Tani et al., 2014; Andersen et al., 2021b). Given the involvement of astrocytes in AD pathology, the importance of brain BCAA metabolism for neurotransmitter homeostasis, and the energetic crisis of AD, it is important to functionally investigate astrocyte BCAA metabolism in relation to AD pathology.

The goals of this study were to perform the first functional profiling of BCAA metabolism in human astrocytes and identify metabolic alterations in BCAA metabolism in the pathophysiology of AD using human induced pluripotent stem cell-derived astrocytes and neurons. First, we demonstrate that astrocytes of acutely isolated human brain slices as well as astrocytes derived from hiPSCs are capable to oxidatively metabolizing the carbon skeleton of BCAAs, particularly for the synthesis of glutamine. Furthermore, astrocytes with mutations in the amyloid precursor protein (APP) or presenilin-1 (PSEN-1) genes known to result in familial forms of AD exhibited decreased synthesis of glutamate, glutamine, and aspartate derived from leucine metabolism. These results uncover highly active astrocytic BCAA metabolism and suggest a possible neurotransmitter imbalance in AD related to leucine metabolism.

## Materials and Methods

### Materials

The stable isotopes [^15^N]leucine, [^15^N]isoleucine, [^15^N]valine, [U-^13^C]leucine, [U-^13^C]isoleucine, [U-^13^C]valine (all L-isomers of 98% chemical purity) were purchased from Cambridge Isotope Laboratories (Tewksbury, MA, United States). All the other chemicals and reagents used were of purest grade available from regular commercial sources.

### Animals

Male NMRI mice were purchased from Harlan (Horst, The Netherlands) and housed at the Department of Drug Design and Pharmacology, University of Copenhagen, in a specific pathogen-free and humidity- and temperature-controlled facility with a 12-h light/dark cycle. The mice were acclimatized for 2 weeks before the experiments and single-housed in individually ventilated cages with free access to food and water. In total, six 12- to 13-week-old mice were used for the experiments (body weight: 42.4 ± 0.4 g). The experiments were approved by the Danish National Ethics Committee and performed according to the European Convention (ETS 123 of 1986).

### Brain Tissue

Human neocortical tissue was obtained from six patients (four females, two males) aged 25–52 years. Additional information on patient cohort characteristics can be found in Andersen et al. (2020). The use of human neocortical tissue was approved by the local Ethics Committee in Copenhagen (H-2-2011-104) with written informed consent from all the patients prior to surgery. Human neocortical tissue of the temporal lobe was resected at Rigshospitalet (Copenhagen, Denmark) in order to facilitate access to the mesial temporal lobe, e.g., amygdala, hippocampus, and parahippocampal gyrus, involved in generating epileptic seizures. Immediately after resection, the tissue was transferred to ice-cold artificial cerebrospinal fluid (ACSF) containing (in mM): NaCl 128, NaHCO_3_ 25, D-glucose 10, KCl 3, CaCl_2_ 2, MgSO_4_ 1.2, and KH_2_PO_4_ 0.4 with pH = 7.4, and transported on ice to the laboratory. Histopathological examination of the neocortical tissue revealed no abnormal pathological features for any of the patients. The metabolic integrity of resected cerebral cortical human tissue has been established previously (Andersen et al., 2020).

### Cell Lines

Human-induced pluripotent stem cell lines were plated on Matrigel-coated plates (BD Matrigel; STEMCELL Technologies, Canada Inc.) with an E8 essential culture medium (STEMCELL Technologies, Canada Inc.). A fresh E8 medium was added every day until the cells reached 90–100% confluency, usually achieved after 7 days of culturing. The hiPSCs used in this study have been previously characterized by Frederiksen et al. (2019a,b) where specific pathogenic mutations in the amyloid precursor protein (APP) or presenilin-1 (PSEN-1) gene were introduced into a healthy iPSC line obtained from the skin biopsy of a healthy person by CRISPR-Cas9. The APP cell line contains a heterozygous double KM670/671NL mutation resulting in an amino acid change from lysine (K) and methionine (M) to asparagine (N) and leucine (L) into the APP gene. The PSEN-1 cell line contains a homozygous E280A mutation resulting in an amino acid change from glutamic acid (E) to alanine (A) into the PSEN-1 gene (Frederiksen et al., 2019a,b). Nucleotide substitution was confirmed by restriction digest and followed by DNA sequencing. Pluripotency of the gene-edited lines was confirmed by immunocytochemistry and quantified by flow analysis. The hiPSC lines used in this study will be referred to as AD astrocytes or AD neurons. These mutations are hallmarks of familial forms of AD with early onset. The healthy hiPSC line was used as the parental control (wild type).

### Neural Progenitor Cell Generation and Cell Differentiation

Neural induction was achieved by dual SMAD inhibition using LDN193189 (S2618, Selleck Chemicals, Houston, TX, United States) and SB431542 (S1067, Selleck Chemicals, Houston, TX, United States), inhibiting the BMP and TGFß pathways, respectively, through a three-dimensional (3D)-sphere method (Chandrasekaran et al., 2017). After 7 days of induction, neuronal progenitor cells (NPCs) were plated on Matrigel in a medium supplemented with basic fibroblast growth factor (bFGF) (10 ng/ml) (CYT-557; ProSpec, Rehovot, Israel) and EGF (10 ng/ml) (CYT-217; ProSpec, Rehovot, Israel). Neuronal rosettes were selected for further passages.

### Astrocytic Differentiation

The astrocytes were differentiated based on a modified protocol by Shaltouki et al. (2013), described elsewhere (Salcedo et al., 2021). Briefly, when the NPCs reached 70% confluency, they were plated on Matrigel in an astrocytic maintenance medium (AMM), which promotes the generation of astrocyte progenitor cells (APCs). To switch from neurogenesis to gliogenesis, the APCs were re-plated several times until passage five. The APCs (last passage) were plated at a seeding density of 50,000 cells/cm^2^ in an astrocytic differentiation medium (ADM). The ADM was changed every other day, and the cells were kept under differentiation conditions for 7 weeks until they reached maturation for metabolic assays.

### Neuronal Differentiation

The neurons were differentiated according to Zhang et al. (2017). When the NPCs reached 70% confluency, they were plated on Matrigel in a neural expansion media (NEM). The NEM was supplemented with 20 ng/ml of bFGF, and EGF. The NPCs were re-plated several times until passage three. Then, they (for last passage) were plated on 0.001% Poly-L-Ornithine (P4957; Sigma-Aldrich, St. Louis, MO, United States) and a 5 μg/ml laminin (L2020; Sigma-Aldrich, St. Louis, MO, United States) coating in a neural maturation media (NMM) for 10 days. The NMM was supplemented with 20 ng/μl brain-derived neurotrophic factor (BDNF) (CYT-207; Prospec, Rehovot, Israel), 10 ng/μl glial cell line-derived neurotrophic factor (GDNF) (CYT-305; Prospec, Rehovot, Israel), 200 μM L-ascorbic acid 2-phosphate (A8960, Sigma-Aldrich, St. Louis, MO, United States), and 50 μM dibutyryl-cyclic adenosine monophosphate (db-cAMP) (A6885; Sigma-Aldrich, St. Louis, MO, United States). Half media change was performed every 3 days. Ten-day immature neurons were passaged a last time on PLO-Laminin coated plates with the NMM at a seeding density of 50,000 cells/cm^2^ until they reached maturation for the metabolic assays.

### Brain Slice Incubations

Incubation of acutely isolated cerebral cortical brain slices of mice and humans was performed as described previously (Andersen et al., 2019, 2020). For mouse brain slices, the experiments were performed one mouse at a time. The mouse was euthanized by cervical dislocation and decapitated. The brain was quickly excised from the cranial vault and submerged in slushed ice-cold ACSF. Cerebral cortices of the mouse brain were dissected, and the rest of the brain was discarded. The isolated mouse cerebral cortices or human neocortical tissue was sliced (350 μm) on a McIlwain tissue chopper (The Vibratome Company, O'Fallon, MO, United States), and slices were separated under a microscope. Two to six mouse cerebral cortical slices or one human slice (gray matter only) was kept just below the surface of 10 ml 37°C oxygenated (5% CO_2_/95% O_2_) ACSF and pre-incubated for 60 min to recover from slicing in a custom-made incubation apparatus (McNair et al., 2017). Subsequently, the media was exchanged for ACSF containing 2 mM ^15^N or ^13^C-labeled leucine, isoleucine, or valine, with a D-glucose concentration of 5 mM, for additional 60 min. Incubations were terminated by transferring the slices to ice-cold 70% ethanol. The slices were sonicated, centrifuged (20,000 *g* × 20 min), and the supernatant was lyophilized before gas chromatography–mass spectrometry (GC–MS) and high-performance liquid chromatography (HPLC) analysis. Protein content of the pellet was determined by the Pierce method using bovine serum albumin (BSA) as standard protein.

### Cell Culture Incubations

Cultures of 7-week-old human-induced pluripotent stem cell-derived astrocytes and neurons were used for dynamic metabolic mapping. The culturing medium was removed, and the cells were washed twice with phosphate-buffered saline (PBS) at 37°C. The cells were then incubated for 90 min at 37°C in ACSF containing 2 mM [U-^13^C]valine, [U-^13^C]leucine, or [U-^13^C]isoleucine plus 2.5 mM unlabeled glucose. These BCAA concentrations were chosen based on previous studies (Andersen et al., 2019). The glucose concentration represents physiological conditions. After incubation, the medium was collected, and the cells were washed with cold PBS (4°C) to stop metabolic reactions. The cells were lysed and extracted with 70% ethanol and centrifuged at 20,000 *g* for 20 min at 4°C to separate soluble and insoluble components. Cell extracts were lyophilized and solubilized in water for further GC-MS and HPLC analysis. Pellets were dissolved in 1 M potassium hydroxide (KOH) at room temperature and analyzed for protein content by the Pierce BSA assay.

### Dynamic Metabolic Mapping by Gas Chromatography–Mass Spectrometry (GC–MS)

The ^15^N and ^13^C-enrichment of TCA cycle metabolites and amino acids was determined by GC–MS analyses, according to a previous method (Walls et al., 2014). Briefly, the extracts were reconstituted in water and acidified, and the metabolites were extracted into an organic phase with 96% ethanol/benzene and derivatized using N-tert-butyldimethylsilyl-N-methyltrifluoroacetamide. Natural ^15^N and ^13^C-abundance was corrected and calculated as described elsewhere (Biemann and McCloskey, 1962). Data are presented as percentage of labeling of the isotopolog M+X, where M corresponds to the molecular weight of the unlabeled molecule, and X is the number of ^15^N or ^13^C-enriched carbon atoms in the molecule (Andersen et al., 2019).

### Quantitative Analysis of Amino Acid Amounts Performed by High Performance Liquid Chromatography (HPLC)

Reconstituted extracts were used for the quantification of amino acid amounts, by reverse phase high-performance liquid chromatography using 1260 Infinity (Agilent Technologies, Sta. Clara, CA, United States), as described elsewhere (Salcedo et al., 2021). Amino acid separation and detection were performed by precolumn o-phthalaldehyde (OPA) online derivatization and fluorescent detection (λex = 338 nm, 10-nm bandwidth; λem = 390 nm, 20-nm bandwidth). A gradient elution with Mobile phase A (10 mM NaH_2_PO_4_, 10 mM Na_2_B_4_O_7_, 0.5 mM NaN_3_, pH 6.8) and mobile phase B (acetonitrile 45%: methanol 45%: H_2_O 10% V:V:V) was performed with a flow of 1.5 ml/min. The quantification of the amino acid amounts was achieved using calibration curves of external standards of the amino acids of interest with known increasing concentration ranging from 5 to 1,000 μM.

### Statistical Analyses

Data are presented as mean (±) standard error of the mean (SEM) of values. Experimental values from the brain tissue were biological replicates, and values from cell cultures were obtained from three independent experiments derived from at least two separate cell differentiations (batches) of three different cell lines, namely the parental control cell line and the two cell lines with the APP- and PSEN-1-introduced mutations, respectively. Statistically significant differences were set at *p* < 0.05 and tested by either one-way or two-way ANOVA with Bonferroni multiple comparison test or with paired *t*-test, as indicated in Figure legends.

## Results

### BCAA Nitrogen Metabolism in Human and Mouse Cortical Slices

**Figure 2:**
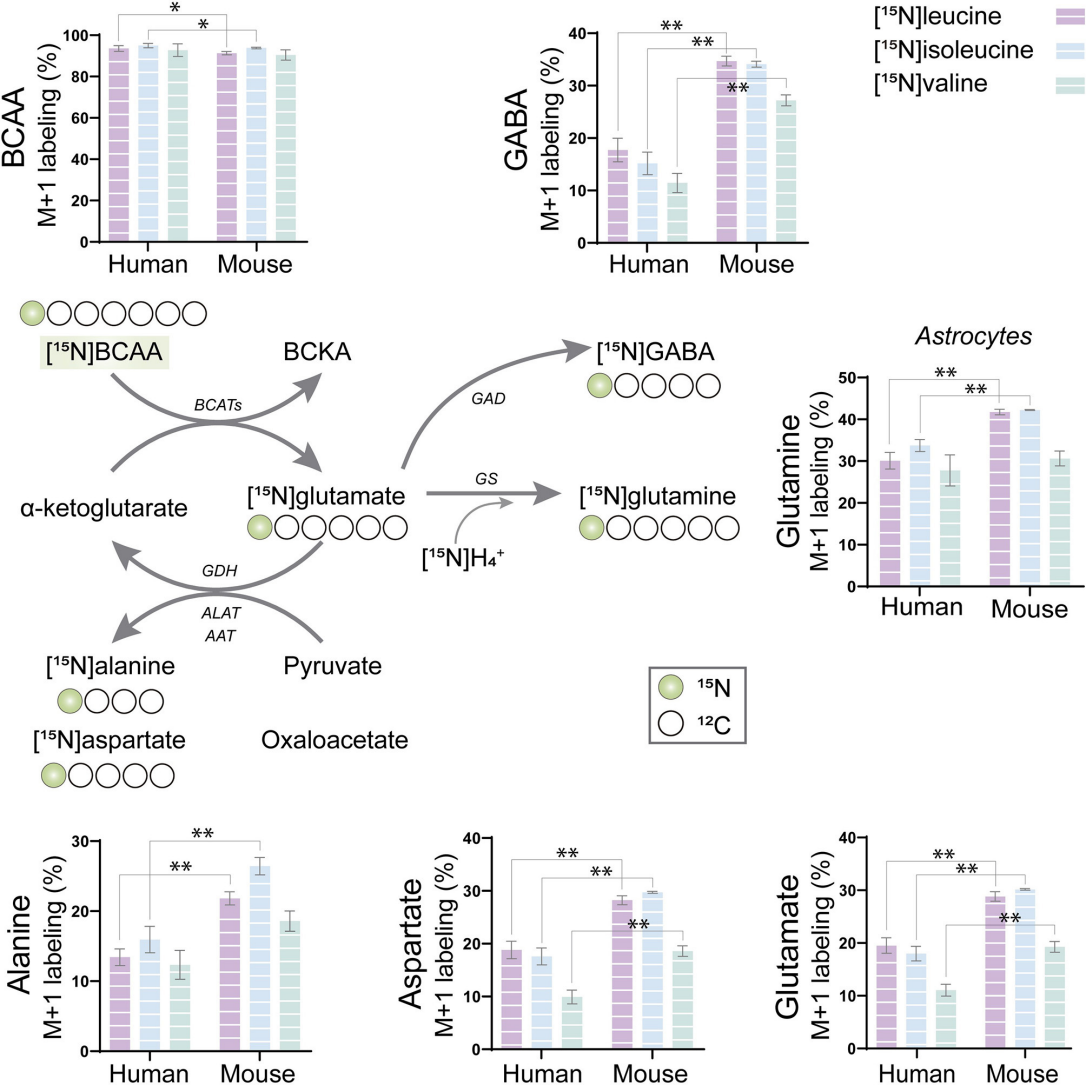
BCAA nitrogen metabolism of human and mouse brain slices. After incubation with [^15^N]BCAA (valine, leucine, or isoleucine), M+1 labeling was observed in glutamate, glutamine, gamma-aminobutyric acid (GABA), alanine, and aspartate, indicating significant nitrogen incorporation into amino acids supported by BCAA metabolism. ^15^N-enrichment in intracellular amino acids was determined by gas chromatography–mass spectrometry (GC-MS). Values represent mean (±) standard error of the mean (SEM) (*n* = 6), ^*^p < 0.05, ^**^p < 0.01 (when compared with human cortical slices) analyzed by Student's paired *t*-test. BCAA, branched-chain amino acid; BCKA, branched-chain α-keto acids; BCATs, branched-chain aminotransferase; GAD, glutamate decarboxylase; GDH, glutamate dehydrogenase; ALAT, alanine aminotransferase; AAT, aspartate aminotransferase; GS, glutamine synthetase.

The BCAAs, leucine, isoleucine, and valine, have been widely described as key nitrogen donors vital for neurotransmission and aid to maintain nitrogen balance in the brain (Conway and Hutson, 2016; Sperringer et al., 2017). The transamination of BCAAs is catalyzed by branched-chain aminotransferase (BCAT) transferring the BCAA nitrogen into glutamate. The nitrogen can subsequently be transferred to connected amino acids. To assess the nitrogen metabolism of the BCAAs, mouse and human cerebral cortical slices were incubated with [^15^N]leucine, [^15^N]isoleucine, and [^15^N]valine. The labeling patterns from the human tissue were compared with the ones obtained from mouse cortical slices in order to distinguish specie-specific functional metabolic differences among cellular compartments (Figure 2). Substantial ^15^N-enrichment was found for all three BCAAs in both mouse and human slices, suggesting a large BCAA uptake capacity. BCAA ^15^N-incorporation was slightly lower in the mouse cortical slices after incubation with [^15^N]leucine and [^15^N]isoleucine when compared with that of the human slices. Increased ^15^N-incorporation was observed in gamma-aminobutyric acid (GABA), aspartate, and glutamine after incubation with [^15^N]leucine, [^15^N]isoleucine, and [^15^N]valine in the mouse cortical slices when compared with the human cortical slices. Furthermore, ^15^N-incorporation in glutamine and alanine was increased after incubation with [^15^N]leucine and [^15^N]isoleucine in the mouse cortical slices. These results may suggest a higher BCAT activity in the mouse brain compared with the human brain. Interestingly, of all the amino acids, the ^15^N-enrichment was highest in glutamine for all the three [^15^N]BCAAs in both mouse and human cortical slices. This observation could indicate that a large proportion of BCAA nitrogen metabolism is being utilized for glutamine synthesis in astrocytes.

### BCAA Oxidative Metabolism in Human and Mouse Cortical Slices

**Figure 3:**
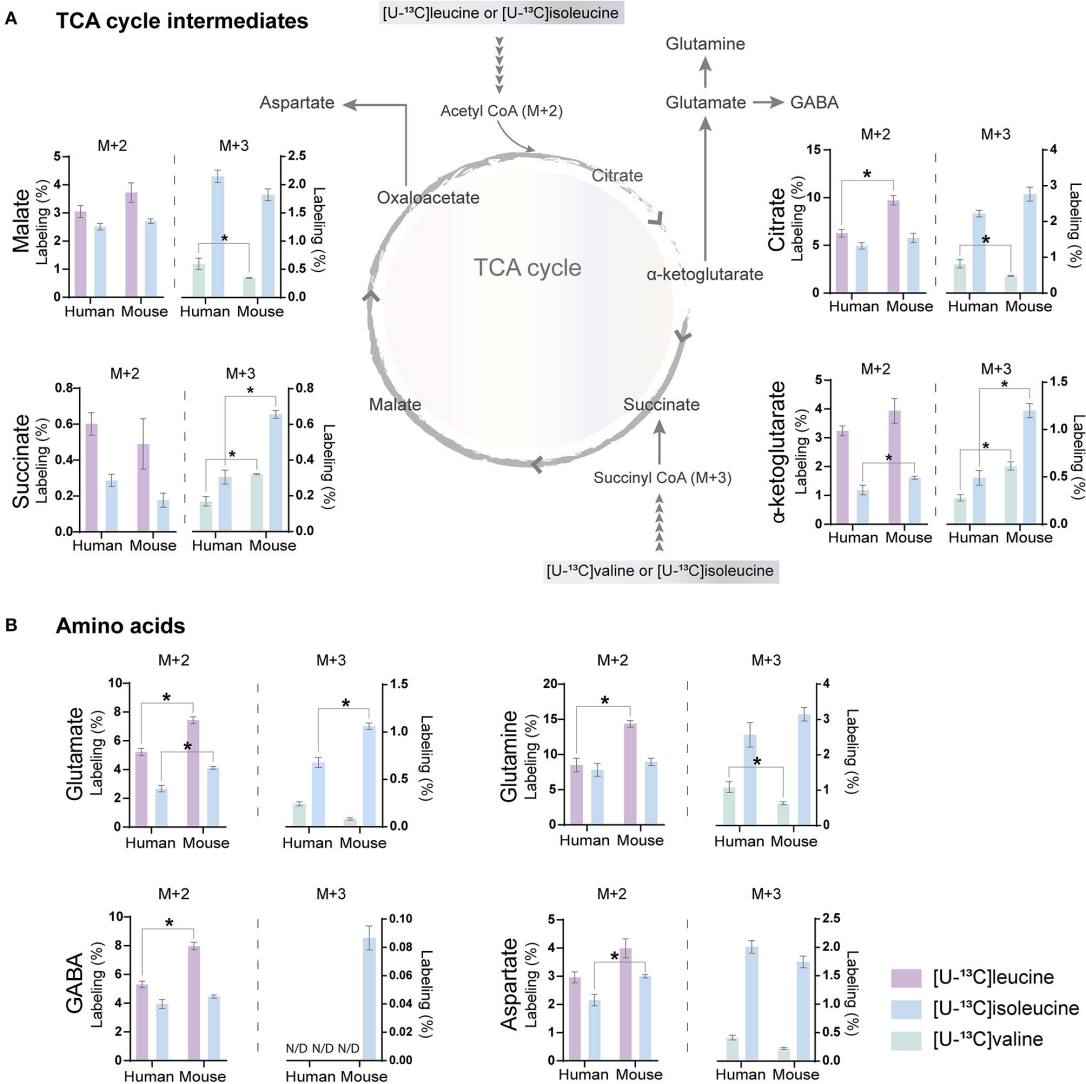
BCAA oxidative metabolism in human and mouse brain slices. ^13^C-enrichment of tricarboxylic acid (TCA) cycle metabolites and amino acids in human and mouse cerebral cortical slices after incubation with [U-^13^C]isoleucine, [U-^13^C]leucine, or [U-^13^C]valine. M+2 and M+3 ^13^C-enrichment in **(A)** TCA cycle intermediates. Increased M+2 ^13^C-enrichment in **(B)** glutamate, glutamine, and GABA after incubation with [U-^13^C]leucine in mouse cortical slices when compared with human cortical slices. ^13^C-enrichment in intracellular metabolites was determined by GC-MS. Values represent mean (±) SEM (*n* = 6), ^*^p < 0.05 analyzed by two-way ANOVA. N/D, not detectable.

The carbon skeleton of the branched-chain keto acid derived from BCAAs can enter the TCA cycle as either acetyl CoA or succinyl CoA (illustrated in Figure 1). The metabolic oxidation of BCAAs is initiated by BCKDH complex activity where irreversible oxidative decarboxylation of the BCKAs occur and *via* multiple subsequent enzymatic reactions yield acetyl CoA or succinyl CoA. To assess BCAA oxidative metabolism, the human and mouse cortical slices were incubated with [U-^13^C]isoleucine, [U-^13^C]leucine, or [U-^13^C]valine, and ^13^C-enrichment of TCA cycle intermediates and amino acids were determined by GC–MS (Figure 3) and amino acid amounts quantified by HPLC (Supplementary Table 1). The metabolism of [U-^13^C]leucine and [U-^13^C]isoleucine (M+6) (Figure 1A) will result in acetyl CoA M+2, whereas the metabolism of [U-^13^C]valine (M+5) and [U-^13^C]isoleucine (M+6) will result in succinyl CoA M+3 (Figure 1B).

Following incubation with [U-^13^C]leucine, ^13^C-enrichment in M+2 (*via* acetyl CoA) was higher in citrate (Figure 3A), glutamate, glutamine, and GABA (Figure 3B) in the mouse cortical slices when compared with the human cortical slices. When incubating with [U-^13^C]isoleucine, we observed increased M+3 labeling (*via* succinyl CoA) in α-ketoglutarate, succinate, and glutamate in the mouse cortical slices when compared with the human cortical slices. Similarly, after incubation with [U-^13^C]valine, ^13^C-incorporation was higher in α-ketoglutarate and succinate in the mouse cortical slices. Decreased ^13^C-incorporation in malate, citrate, and glutamine was observed in the mouse cortical slices when compared with the human cortical slices after incubation with [U-^13^C]valine. The overall highest ^13^C-enrichment was derived from [U-^13^C]leucine metabolism in both the mouse and human cortical slices. In accordance with the results of [^15^N]BCAA metabolism, glutamine and citrate reached the highest ^13^C-enrichment after incubation with all the three [U-^13^C]BCAAs in both the mouse and human cortical slices. These results demonstrate that oxidative metabolism of the BCAA carbon skeleton is utilized for amino acid synthesis, particularly for glutamine synthesis in astrocytes from both the mouse and human cerebral cortical slices.

### BCAA Oxidative Metabolism in Human Astrocytes

**Figure 4:**
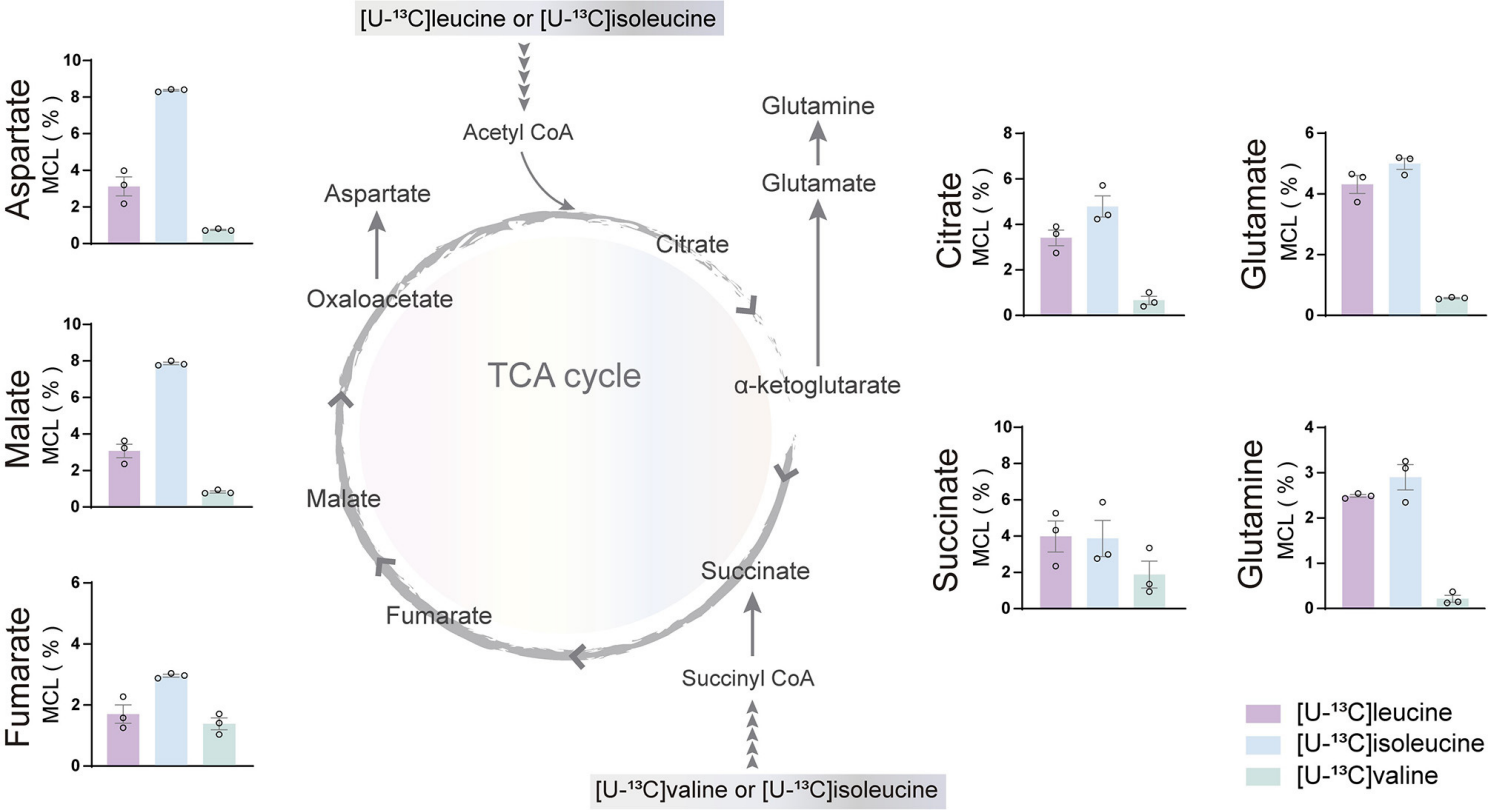
BCAA oxidative metabolism in human induced pluripotent stem cell (hiPSC)-derived astrocytes. hiPSC-derived astrocytes were incubated with [U-^13^C]isoleucine, [U-^13^C]leucine, or [U-^13^C]valine. Labeling in TCA cycle intermediates and amino acids represented as molecular carbon labeling (MCL) confirms hiPSC-derived astrocytes capability to oxidatively metabolize the BCAA isoleucine, leucine, and, to a lesser extent, valine by incorporating them in the TCA cycle. Values represent mean ± SEM (*n* = 3) from different hiPSC-derived astrocyte set-ups individually depicted as circles.

The incubation experiments revealed that the brain slices of both mouse and humans have a large capacity for BCAA metabolism particularly supporting astrocyte glutamine synthesis. To further substantiate these results, we next assessed if hiPSC-derived astrocytes could metabolize BCAAs through ^13^C-incorporation into the TCA cycle. Incubation with the three [U-^13^C]BCAAs (Figure 4) is presented as the molecular carbon labeling (MCL), which is the average of the carbon labeling in a given molecule (Andersen et al., 2021a). ^13^C-enrichment was recovered in all TCA cycle metabolites and amino acids from [U-^13^C]BCAA metabolism, which demonstrates that human astrocytes are capable of introducing and metabolizing the carbon skeleton of leucine, isoleucine, and valine in the TCA cycle. Interestingly, the MCL obtained from incubation with [U-^13^C]isoleucine appeared to be higher throughout all the TCA cycle intermediates and amino acids compared with leucine and valine. However, as the metabolism of [U-^13^C]isoleucine gave rise to both M+2 and M+3 labeling, the MCL is expected to be higher. [U-^13^C]valine had the lowest overall ^13^C-enrichment in the TCA cycle intermediates and amino acids, which is in accordance with the results from mouse and human brain slices. These results functionally demonstrate that hiPSC-derived astrocytes are able to oxidatively metabolize BCAAs.

### Altered BCAA Oxidative Metabolism in AD hiPSC-Derived Astrocytes With APP or PSEN-1 Mutations

**Figure 5:**
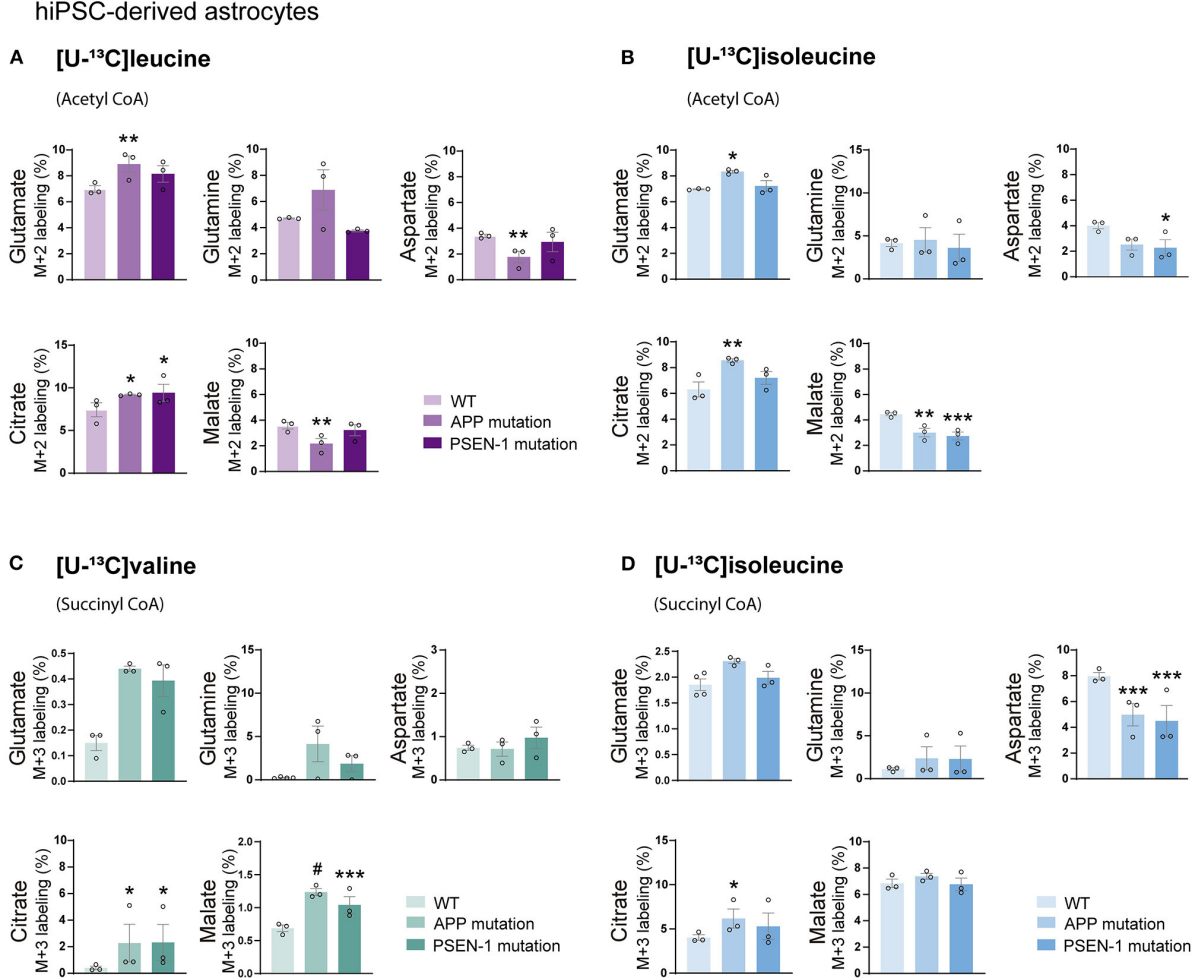
Reduced oxidative BCAA metabolism entering as acetyl CoA in Alzheimer's disease (AD) astrocytes. **(A)** Increased glutamate and citrate M+2 labeling but decreased aspartate and malate ^13^C-enrichment after incubation with [U-^13^C]leucine and **(B)** [U-^13^C]isoleucine in human AD astrocytes when entering *via* acetyl CoA. **(C)** Increased TCA cycle metabolites M+3 labeling after incubation with [U-^13^C]valine but **(D)** decreased aspartate ^13^C-enrichment after incubation with [U-^13^C]isoleucine in human AD astrocytes when entering *via* succinyl CoA. Values represent mean (±) SEM (*n* = 3), ^*^p < 0.05, ^**^p < 0.01, ^***^p < 0.001, ^#^*p* < 0.0001, analyzed by two-way ANOVA. WT, wild type; APP, amyloid precursor protein; PSEN-1, presenilin-1.

Studies on postmortem AD brains have reported alterations in the expression of enzymes involved in the metabolism of BCAAs, such as BCAT (Hull et al., 2015). However, functional BCAA metabolism in relation to AD pathophysiology has not been explored. Given the central metabolic role of BCAAs in neurotransmission, we sought to determine whether BCAA oxidative metabolism is affected in hiPSC-derived astrocytes with APP or PSEN-1 mutation as models of AD pathology (referred to as AD astrocytes). hiPSC-derived astrocytes from either AD-mutated or parental control cell lines were, thus, incubated with [U-^13^C]leucine, [U-^13^C]isoleucine, or [U-^13^C]valine, and ^13^C-incorporation into the TCA cycle intermediates and amino acid were assessed (Figure 5). After incubation with [U-^13^C]leucine, AD astrocytes with APP mutation exhibited increased labeling in glutamate and citrate (M+2) but reduced ^13^C-enrichment was found in aspartate and malate when compared with control astrocytes. The AD astrocytes with PSEN-1 mutation only exhibited increased labeling in citrate (M+2) but otherwise similar labeling patterns when compared with the control astrocytes (Figure 5A). When incubated with [U-^13^C]isoleucine, similar changes between AD and control astrocytes were observed in amino acid labeling (M+2) as observed in the [U-^13^C]leucine incubation (Figure 5B). For instance, in the AD astrocytes with APP mutation, glutamate (M+2) labeling was increased, while labeling in aspartate was decreased although not significantly, compared with the control astrocytes. In the AD astrocytes with PSEN-1 mutation, however, the decrease in aspartate labeling was significantly different from that of the control astrocytes. ^13^C-incorporation in citrate was increased only in the AD astrocytes with APP mutation, while labeling in malate (M+2) was decreased in both AD astrocytes when compared with the control astrocytes. No significant differences were found in the labeling of α-ketoglutarate, succinate, and fumarate (Supplementary Table 2). Furthermore, after incubation with [U-^13^C]valine (Figure 5C), no significant differences between the AD and control astrocytes in amino acid labeling (M+3) were found. However, the TCA cycle metabolites, citrate, and malate showed increased ^13^C-enrichment in both mutated AD astrocytes when compared with control. Following [U-^13^C]isoleucine metabolism, the AD astrocytes only exhibited lower labeling in aspartate (M+3) compared with the control astrocytes (Figure 5D). No significant differences were found in the labeling of α-ketoglutarate, succinate, and fumarate (Supplementary Table 3). Collectively, these results suggest that BCAA oxidative metabolism in AD astrocytes is impaired particularly when the BCAAs enter the TCA cycle *via* acetyl CoA.

### Decreased Aspartate Labeling in AD hiPSC-Derived Neurons With APP or PSEN-1 Mutations After Incubation With BCAAs

**Figure 6:**
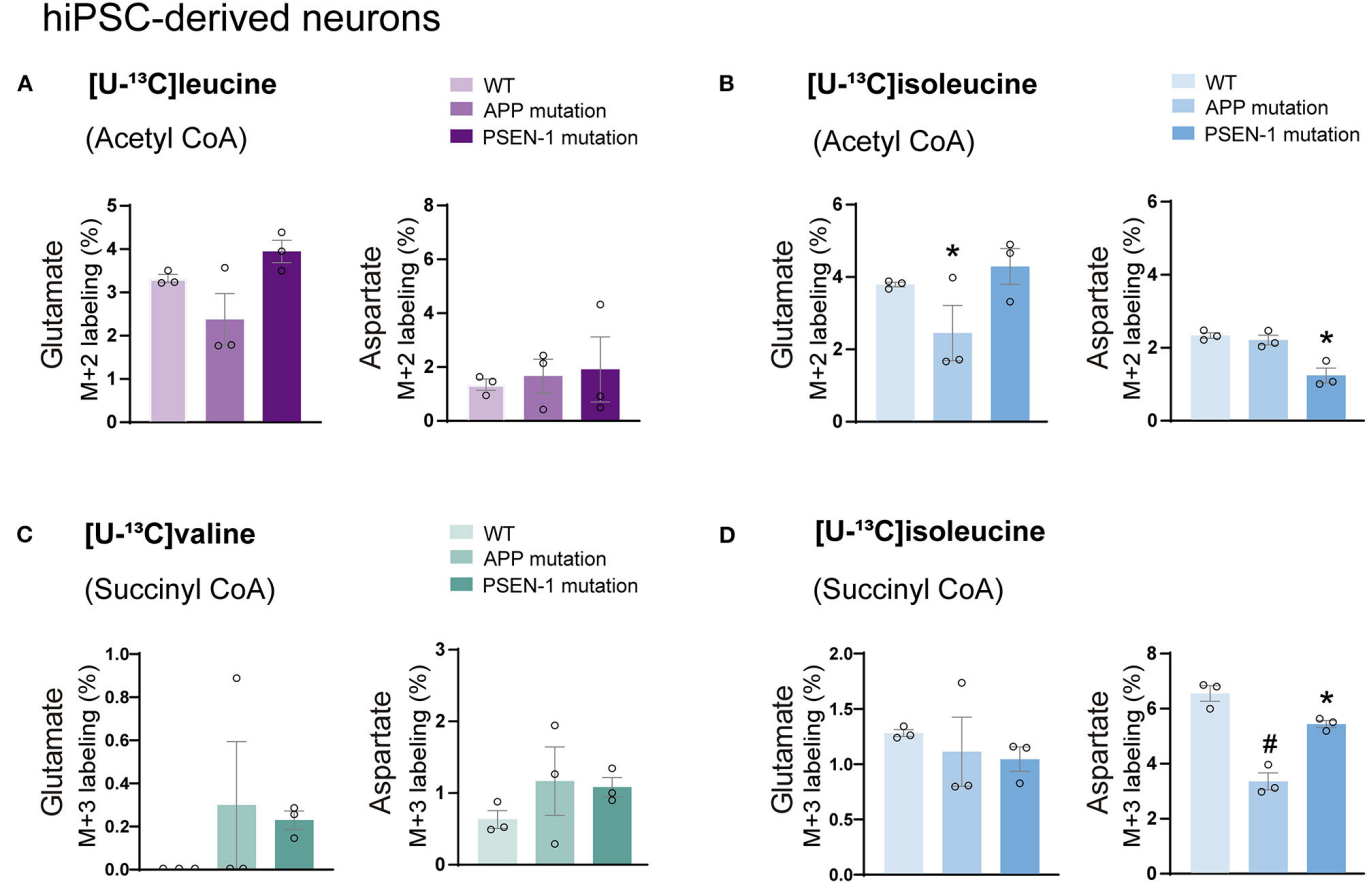
Decreased aspartate synthesis from oxidative isoleucine metabolism in AD neurons. **(A)** No significant differences were found in AD neurons after incubation with [U-^13^C]leucine. **(B)** Decreased glutamate M+2 and aspartate M+2 labeling after incubation with [U-^13^C]isoleucine in AD neurons. **(C)** No significant differences were found in AD neurons after incubation with [U-^13^C]valine in AD neurons. **(D)** Decreased aspartate M+3 labeling after incubation with [U-^13^C]isoleucine in AD neurons when compared with control. No significant differences were found in TCA cycle intermediates. Values represent mean (±) SEM (*n* = 3), ^*^p < 0.05, ^#^*p* < 0.0001 analyzed by two-way ANOVA. WT, wild type; APP, amyloid precursor protein; PSEN-1, presenilin-1.

Astrocytes and neurons maintain tight metabolic interactions critical for cerebral homeostasis. After finding alterations in the BCAA metabolism of AD hiPSC-derived astrocytes, we next assessed if BCAA oxidative metabolism could be affected in hiPSC-derived neurons with APP or PSEN-1 mutation as models of AD pathology (referred to as AD neurons). hiPSC-derived neurons from AD-mutated or parental control cell lines were incubated with [U-^13^C]leucine, [U-^13^C]isoleucine, or [U-^13^C]valine, and ^13^C-incorporation into the TCA cycle intermediates and amino acids was assessed (Figure 6). After incubation with [U-^13^C]leucine, no significant differences were observed in the labeling of glutamate or aspartate (M+2) in the AD neurons compared with the control neurons (Figure 6A). In contrast, following incubation with [U-^13^C]isoleucine (Figure 6B), decreased glutamate labeling (M+2) in the AD neurons with APP mutation and decreased aspartate (M+2) labeling in the AD neurons with PSEN-1 mutation were found compared with the control neurons. No significant differences between the AD and control neurons were observed in glutamate or aspartate labeling after incubation with [U-^13^C]valine (Figure 6C). Finally, reduced labeling in aspartate (M+3) derived from [U-^13^C]isoleucine metabolism (*via* succinyl CoA) was found in both mutated AD neurons when compared with the control neurons (Figure 6D). No significant differences were found in the labeling of citrate, α-ketoglutarate, succinate, fumarate, and malate (Supplementary Tables 4, 5). These results demonstrate decreased ^13^C-incorporation in aspartate (M+2 and M+3) after incubation with [U-^13^C]isoleucine in AD neurons, suggesting that BCAA oxidative metabolism in AD hiPSC-derived neurons is largely maintained in contrast to the AD hiPSC-derived astrocytes.

### Decreased Amino Acids Amounts in AD hiPSC-Derived Astrocytes and Neurons After Incubation With BCAAs

**Figure 7:**
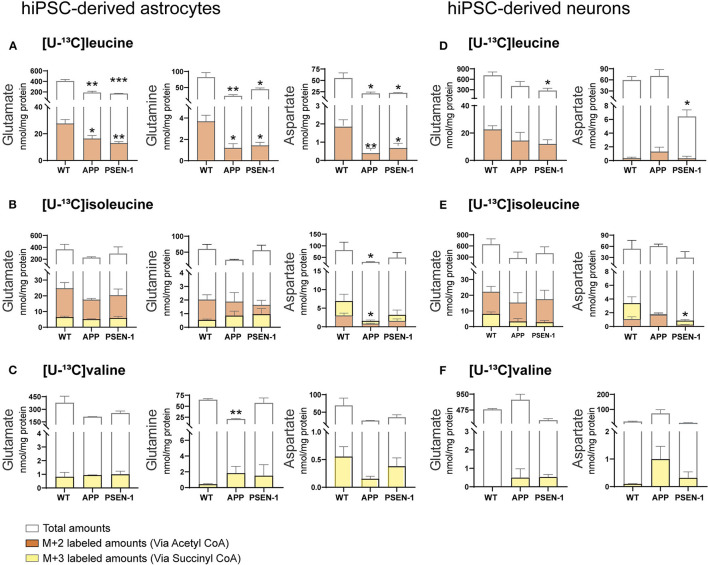
Selective disruptions of amino acid amounts in AD astrocytes with APP and PSEN-1 mutations following incubation with [U-^13^C]leucine. **(A)** nmol/mg protein in M+2 (*via* acetyl CoA) labeling and total amounts after incubation with [U-^13^C]leucine. **(B)** nmol/mg protein in M+2 (*via* acetyl CoA) and M+3 (*via* succinyl CoA) labeling and total amounts after incubation with [U-^13^C]isoleucine. **(C)** nmol/mg protein in M+3 (*via* succinyl CoA) labeling and total amounts after incubation with [U-^13^C]valine in hiPSC-derived astrocytes. **(D)** nmol/mg protein in M+2 (*via* acetyl CoA) labeling and total amounts after incubation with [U-^13^C]leucine. **(E)** nmol/mg protein in M+2 (*via* acetyl CoA) and M+3 (*via* succinyl CoA) labeling and total amounts after incubation with [U-^13^C]isoleucine. **(F)** nmol/mg protein in M+3 (*via* succinyl CoA) labeling and total amounts after incubation with [U-^13^C]valine in hiPSC-derived neurons. Values represent mean (±) SEM (*n* = 3), ^*^p < 0.05, ^**^p < 0.01, ^***^p < 0.001 when compared with control, analyzed by one-way ANOVA. No significant differences were found in M+3 labeling amounts in AD astrocytes. WT, wild type; APP, amyloid precursor protein; PSEN-1, presenilin-1.

Total and labeled amounts of intracellular amino acids from the hiPSC-derived astrocytes and neurons were determined by HPLC. AD hiPSC-derived astrocytes with APP and PSEN-1 mutations showed decreased total and labeled M+2 amounts in glutamate, glutamine, and aspartate after [U-^13^C]leucine-derived carbons entered the TCA cycle *via* acetyl CoA (Figure 7A). As mentioned previously, [U-^13^C]isoleucine-derived carbons can enter the TCA cycle *via* acetyl CoA or succinyl CoA, giving rise to double labeled (M+2) or three labeled (M+3) intermediates, respectively. Labeled M+2 amounts and total amounts of aspartate were lower in the AD astrocytes with APP mutation when compared with the control astrocytes after incubation with [U-^13^C]isoleucine (Figure 7B). In contrast, no differences were observed in labeled M+3 amounts of any amino acid or in the total amounts of glutamate and glutamine in both mutated AD astrocytes when compared with the control astrocytes after [U-^13^C]isoleucine incubation. No significant differences were observed in labeled M+3 amounts of mutated AD astrocytes after [U-^13^C]valine incubation (Figure 7C). However, total amounts of glutamine were lower in the AD astrocytes with APP mutation compared with control.

After incubation with [U-^13^C]leucine, total amounts of glutamate and aspartate (Figure 7D) were decreased only in the AD neurons with PSEN-1 mutation compared with the control neurons. Similarly, the AD neurons with PSEN-1 mutation exhibited decreased amounts of labeled M+3 in aspartate (Figure 7E) after [U-^13^C]isoleucine entry *via* succinyl CoA when compared with control. No significant differences were found in labeled or total amino acid amounts after incubation with [U-^13^C]valine in the AD neurons when compared with the control neurons (Figure 7F). These results confirm that synthesis of the amino acids glutamate, glutamine, and aspartate, derived from leucine metabolism, is impaired to a larger extent compared with isoleucine and valine metabolism in AD hiPSC-derived astrocytes. Lastly, only the synthesis of aspartate derived from leucine and isoleucine metabolism was decreased in the AD hiPSC-derived neurons compared with the control neurons, which indicates that the metabolism of BCAAs is significantly affected in the AD astrocytes but generally maintained in the AD neurons.

## Discussion

**Figure 8:**
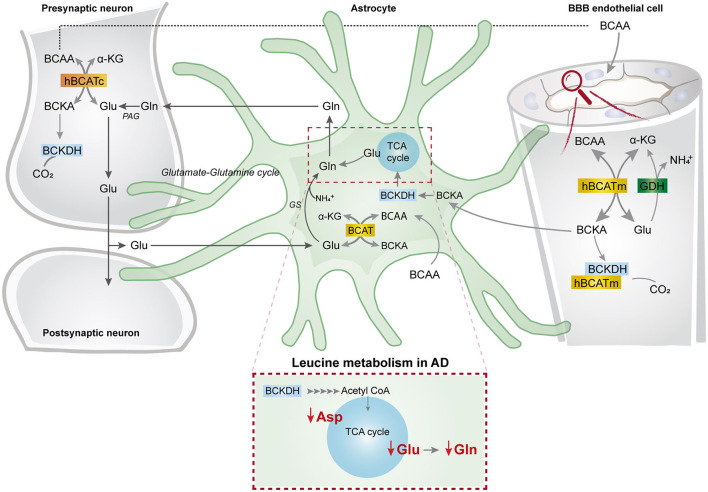
BCAA oxidative metabolism and nitrogen transfer in human astrocytes and potential blood brain barrier-astrocyte-neuron axis. BCAAs are taken up by the blood brain barrier (BBB) endothelial cells, and then BCAAs can undergo transamination by the action of BCATm, forming glutamate and the branched-chain α-keto acids (BCKAs), KIC, KIV, and KVM. In the reverse reaction when α-ketoglutarate is formed from glutamate, the BCAT/GDH metabolon in the endothelial cells may release ammonia, and BCAAs can be released and taken up by neurons or astrocytes. Alternatively, BCKAs from endothelial cells can be released or metabolized by the BCKDH/BCATm metabolon. Likewise, in neurons, BCAAs can also be transaminated, producing BCKAs and glutamate. BCKAs can be further metabolized by BCKDH, and glutamate can be used for neurotransmission. Glutamate from neurons in the synaptic cleft can be taken up by astrocytes. Astrocytes synthesize glutamine from glutamate and ammonia which is catalyzed by glutamine synthetase (GS) and is released for neuronal uptake to replenish the glutamate pool, in the so-called glutamate-glutamine cycle. The data indicate that in astrocytes, BCAAs may be transaminated by the action of BCAT, and that the BCAA carbon skeleton can be oxidized by BCKDH activity. This set of metabolic reactions give rise to acetyl CoA or succinyl CoA, replenishing TCA cycle intermediates and amino acids in the astrocytes. Furthermore, we present evidence of reduced aspartate, glutamate, and glutamine total and labeling amounts after incubation with [U-^13^C]leucine in AD astrocytes (represented within the dotted box). This altered leucine metabolism in AD astrocytes may contribute to the disrupted neurotransmitter homeostasis observed in the AD brain. BBB, blood brain barrier; BCATm, mitochondrial branched-chain aminotransferase; Glu, glutamate; α-KG, α-ketoglutarate, GDH, glutamate dehydrogenase, ${\text{NH}}_{4}^{+}$, ammonium; BCKA, branched-chain α-keto acids; KIC, α-ketoisocaproate; KIV, α-ketoisovaleric; KMV, α-keto-β-methylvalerate; BCAT, branched-chain aminotransferase; BCKDH, branched-chain α-keto acid dehydrogenase; Gln, glutamine; GS, glutamine synthetase; BCATc, cytosolic branched-chain aminotransferase; PAG, phosphate-activated glutaminase.

This study represents the first functional evidence of active BCAA metabolism in human astrocytes. We found that the metabolism of the BCAA carbon skeleton supported astrocyte glutamine synthesis in acutely isolated human brain slices, which was also observed in hiPSC-derived astrocytes. Interestingly, we found reduced synthesis of amino acids derived from leucine metabolism in AD hiPSC-derived astrocytes when compared with the control astrocytes. Furthermore, the synthesis of glutamate and aspartate derived from leucine metabolism was decreased in the AD neurons with PSEN-1 mutation. Our findings are summarized in Figure 8 along with a new proposed model of BCAA nitrogen and oxidative metabolism in the human brain.

### BCAA Metabolism in Mouse and Human Brain Slices

BCAA metabolism is important for cerebral nitrogen homeostasis and neurotransmitter recycling, as these amino acids are nitrogen donors for glutamate and GABA synthesis, whereas the carbon skeleton can be utilized as oxidative fuels (Yudkoff, 1997; Conway and Hutson, 2016). From incubations of acutely isolated brain slices, we found that brain slices of both mice and humans have a large capacity for BCAA nitrogen and oxidative metabolism (Figures 2, 3). Generally, we observed a higher fractional enrichment of both ^15^N and ^13^C in the brain slices of mice when compared with human slices, which may suggest that the rodent brain have a higher capacity for BCAA metabolism. Interestingly, immunohistochemistry studies have suggested that the cellular distribution of enzymes related to BCAA metabolism differs between the rodent and human brains (Sperringer et al., 2017). Specific isoforms of the initiating enzyme of BCAA metabolism, BCAT, have been located in both neurons (BCAT1 corresponding to BCATc) and astrocytes (BCAT2 corresponding to BCATm) in the rodent brain (Sweatt et al., 2004), whereas BCKDH was only found in neurons (Cole et al., 2012). This contrasts with the reported expression in the human brain, where both the BCAT and BCKDH expressions were found in neurons and endothelial cells, but were absent in astrocytes (Hull et al., 2012, 2018). These studies imply that both mouse and human astrocytes have a limited enzymatic capacity for BCAA metabolism. Particularly, the complete absence of the rate-limiting enzyme BCKDH in astrocytes would imply that this cell type is unable to oxidatively metabolize the BCAA carbon skeleton in the TCA cycle. However, multiple studies using mouse astrocyte cultures have functionally shown that astrocytes are able to metabolize BCAAs (Yudkoff et al., 1996; Johansen et al., 2007; Murín et al., 2009a,b). Although most of the carbon skeleton may be released as the corresponding ketoacid or as ketone bodies (Bixel and Hamprecht, 1995; Yudkoff et al., 1996), a significant fraction enters the astrocytic TCA cycle, which will require astrocyte BCKDH activity. A recent proteomic study resolving the cellular protein expression of the mouse brain confirmed the differential expression BCATc in neurons and BCATm in astrocytes (Sharma et al., 2015). However, the same study found higher expression of the two BCKDH isoforms (BCKDHA and BCKDHB) in both cultured and isolated astrocytes when compared with neurons (Sharma et al., 2015). In the brain slices, we found the largest fractional ^13^C-enrichment from ^13^C-BCAA metabolism in glutamine (Figure 3), which is in line with a recent study by the group of the authors in a mouse model of Huntington's disease (Andersen et al., 2019). Since glutamine is exclusively synthesized in astrocytes, because of the selective expression of the enzyme glutamine synthetase (GS) (Norenberg and Martinez-Hernandez, 1979), the large ^13^C-enrichment in glutamine from BCAA metabolism strongly indicates active astrocyte BCAA oxidative metabolism. Furthermore, of the TCA cycle intermediates, citrate displayed the highest ^13^C-enrichment from ^13^C-BCAA metabolism, which is also an indicator of astrocyte metabolism in acute brain slices (Andersen et al., 2017; McNair et al., 2017). Finally, we also characterized oxidative BCAA metabolism in cultured human iPSC-derived astrocytes, where we likewise observed a conserved capacity for BCAA oxidative metabolism (Figure 4). Hence, the functional metabolic analyses conflict with the previous immunohistochemical investigations, and demonstrate active BCAA metabolism in both mouse and human astrocytes. The discrepancy between the functional metabolic studies and the immunohistochemical studies may be related to methodological limitations of immunohistochemistry, as discussed in (Danbolt et al., 2016).

### BCAA Metabolism in AD

Branched-chain amino acid metabolism has been described to be altered in several neurological disorders. These alterations can arise from deficiencies in the expression and function of metabolic enzymes as observed in Maple syrup urine disease, which is caused by deficiency in BCKDH and leads to severe neurological symptoms (Chuang and Chuang, 2000). Interestingly, decreased plasma and brain concentrations of BCAAs have consistently been reported in patients with Huntington's disease (HD), which correlates with disease severity (Perry et al., 1972; Mochel et al., 2011). Using a mouse model of HD, we have previously reported an elevated capacity of BCAA metabolism in cerebral cortical and striatal slices (Andersen et al., 2019). The enhanced BCAA metabolism was related to an increased expression of BCAA metabolic proteins and suggests that the reduced BCAA concentrations in HD may be due to compensatory increases in BCAA brain metabolism (Andersen et al., 2019). Evidence from both disease models and patients strongly indicate involvement of BCAA metabolism in AD pathology. For instance, it was found in patients with AD that genetic predisposition to elevated isoleucine plasma levels, but not leucine or valine, is positively associated with AD (Larsson and Markus, 2017). Furthermore, a large reduction in the concentration of valine has been detected in the cerebrospinal fluid (Basun et al., 1990) and plasma (González-domínguez et al., 2015; Toledo et al., 2017) of patients with AD patients, and the expression of hippocampal BCATc in patients with AD was increased by 28% compared with control brains (Hull et al., 2015). Interestingly, elevated BCAA levels have been found in the APP/PS1 mouse model of AD (Ruiz et al., 2016). BCAA supplementation has been shown to be beneficial in disorders with systemic elevated ammonia levels, e.g., hepatic encephalopathy (Holecek, 2018). In the periphery, BCAAs stimulate glutamine synthesis in muscles, hereby fixating ammonia (Holecek et al., 2011). In the brain, glutamine synthesis is also the primary pathway of ammonia fixation and is strictly located in astrocytes (Norenberg and Martinez-Hernandez, 1979; Brusilow et al., 2010). Interestingly, AD has been associated with elevated cerebral levels of ammonia (Seiler, 2002), which may be caused by insufficient astrocyte glutamine synthesis (Smith et al., 1991; Olabarria et al., 2011; Andersen et al., 2021a). Since glutamine is derived from the TCA cycle intermediate α-ketoglutarate *via* glutamate, astrocyte TCA cycle function is crucial for glutamine synthesis (Swanson and Graham, 1994). Multiple studies have revealed impaired astrocytic energy metabolism in AD (Oksanen et al., 2017; Dematteis et al., 2020; Andersen et al., 2021a; Ryu et al., 2021). It could, therefore, be speculated that the reduced synthesis of glutamate from oxidative leucine metabolism observed in the AD astrocytes may be caused by deficient astrocyte TCA cycle function, which may further hamper glutamine synthesis in AD. Furthermore, ammonia has been shown to inhibit oxidative astrocyte metabolism (Lerchundi et al., 2015), potentially further reducing glutamine synthesis. Further studies are needed to explore the potential link of disrupted ammonia homeostasis and BCAA metabolism in AD.

Here we found that in the AD astrocytes, the TCA cycle oxidation of leucine, isoleucine, and valine was largely maintained (Figure 5). However, citrate from metabolism of [U-^13^C]leucine, [U-^13^C]isoleucine, and [U-^13^C]valine displayed increased ^13^C-enrichment in AD astrocytes. It has been proposed that the astrocytic release of citrate may modulate neuronal excitability through the regulation of extracellular concentrations of Ca^2+^ and Mg^2+^ (Westergaard et al., 1994a,b). Furthermore, disruptions in brain citrate levels can alter neuronal excitability in the hippocampus, resulting in epileptic seizures (Henke et al., 2020). The results of increased citrate labeling from BCAA metabolism may reflect a supportive role of citrate transfer from astrocytes to neurons (Hertz et al., 1992).

BCAA metabolism is closely associated to the fate of glutamate. Glutamate acts as the link between neurotransmission and energy metabolism (Mckenna et al., 1996, 2019; Mckenna, 2013). Glutamate synthesis in astrocytes occurs mainly as transamination, particularly *via* aspartate aminotransferase (AAT) (Westergaard et al., 1996). The conversion of α-ketoglutarate to glutamate may depend on nitrogen from BCAAs, primarily leucine, *via* BCAT activity. It has been suggested that at least 20% of all glutamate nitrogen is derived from leucine in cultured astrocytes (Yudkoff et al., 1994). Interestingly, the results show reduced amino acid synthesis derived from leucine metabolism in AD astrocytes (Figure 7). Since AD astrocytes exhibited decreased amounts of glutamate derived from leucine metabolism, it can be speculated that impaired leucine nitrogen metabolism could reduce glutamate metabolic pools in the AD astrocytes. Leucine is also an efficient nitrogen donor for glutamine synthesis (Yudkoff et al., 1994, 2005). This is in line with the results showing that nitrogen metabolism of BCAAs is mainly utilized for glutamine synthesis in human and mouse cortical slices. Since glutamine is synthesized from glutamate, it can be suggested that the observed alterations in the glutamate pools could have a direct influence on glutamine synthesis, as observed in AD astrocytes. Metabolism of the BCAA carbon skeleton will result in net transfer of nitrogen to nonessential amino acids (Conway and Hutson, 2016), with the possibility of replenishing neurotransmitter pools. Interestingly, we found decreased amounts of labeling in amino acids derived from the oxidative metabolism of leucine in AD astrocytes. This reduction in amino acid synthesis derived from leucine metabolism, particularly in glutamine, could influence neurotransmitter recycling and homeostasis in AD astrocytes. Furthermore, one-third of the ^13^C-incorporation from leucine metabolism will give rise to M+1 labeling in TCA cycle intermediates and amino acids. AD astrocytes, not AD neurons, displayed altered M+1 labeling (Supplementary Table 6). The labeling of succinate, malate, and aspartate (M+1) from leucine metabolism was reduced in the AD astrocytes with APP mutation. However, citrate (M+1) labeling was increased in both mutated AD astrocytes. These results further confirm that oxidative leucine metabolism is altered in AD astrocytes but maintained in AD neurons.

We found that glutamate labeling amounts from the metabolism of BCAAs in AD neurons were unaltered (Figure 7). However, glutamate and aspartate total amounts from leucine metabolism were decreased in the AD neurons with PSEN-1 mutation. BCAT isoforms are redox-sensitive proteins and are in their reduced form part of a metabolon with glutamate dehydrogenase (GDH) catalyzing the conversion of glutamate into α-ketoglutarate (Conway et al., 2002, 2004, 2008). An increase in oxidative stress (OS) might change the redox status, preventing this metabolon formation (Harris et al., 2020). The reduced glutamate and aspartate total amounts observed in AD neurons may be due to changes in the redox state of BCAT, causing reduced amino acid synthesis. Furthermore, leucine is a positive allosteric modulator of GDH activity (Li et al., 2011), and changes in leucine amounts may hereby perturb glutamate homeostasis *via* GDH activity in AD. Imbalanced redox states, caused by an excessive accumulation of reactive oxygen species (ROS) and a decrease in antioxidant enzymes, leads to OS in AD (Zhao and Zhao, 2013; Wang et al., 2014; Kim et al., 2015). Alterations in glutathione (GSH), the most abundant brain antioxidant, have been described to be reduced in patients with AD (Mandal et al., 2012, 2015; Saharan and Mandal, 2014). In several studies using different AD models, OS can also be induced by Aβ accumulation (Pappolla et al., 1998; Butterfield, 2002; Cheignon et al., 2018), which could eventually lead to mitochondrial dysfunction (Zhao and Zhao, 2013; Wang et al., 2014). Alterations in the mitochondria might represent the major source of OS observed in AD (Castellani et al., 2002; Wang et al., 2014), since the majority of endogenous ROS is produced by the mitochondria (Wang et al., 2014). These alterations in OS could contribute further to mitochondrial impairments, such as metabolic imbalances.

Branched-chain amino acids also have a regulatory role in the mammalian target of rapamycin (mTOR) pathway. The mTOR pathway is an environmental sensor, which regulates cell proliferation, protein synthesis, and autophagy through multiple signaling pathways (Jewell et al., 2015; Shafei et al., 2017; Liu and Sabatini, 2020). mTOR constitutes the catalytic subunit of two distinct complexes (Shafei et al., 2017). mTOR complex 1 (mTORC1) plays a role in neuronal synaptic plasticity and phosphorylates substrates to promote synthesis of proteins, lipids, nucleotides, etc., while repressing the autophagic breakdown of cellular components. mTORC1 activation is regulated by nutrients such as several amino acids, particularly leucine and arginine (Liu and Sabatini, 2020). Depletion of amino acids, low glucose concentrations, and OS can negatively impact mTORC1 regulation while stimulating autophagy (Shafei et al., 2017). Some studies have reported that mitochondrial ROS are increased by high concentrations of BCAAs in human endothelial and peripheral blood cells (Zhenyukh et al., 2017, 2018). However, inhibition of the mTORC1 pathway, decreased BCAAs-induced pro-oxidant effects. Likewise, alterations in mTORC1 regulation in AD pathology may result in accumulation of amyloid-β (Aβ) aggregates. Increased mTOR activity and signaling were found in APP-transfected cell lines and in brains of 3xTg-AD mice (Caccamo et al., 2010, 2011). Interestingly, mTOR activity was reduced when preventing Aβ accumulation in the 3xTg-AD mice. Apparently, high Aβ levels can exert toxicity through increased mTOR activity. In addition, the use of rapamycin ameliorated cognitive deficits, and Aβ and Tau pathology in 3xTg-AD mice by increasing autophagy (Caccamo et al., 2010). It has been suggested that the alteration of mTOR signaling and autophagy occurs at early stages of AD. The status of the mTOR pathway in AD post-mortem brain tissue has been investigated, where increased reduction in autophagy was observed as the disease progressed (Tramutola et al., 2015). Since leucine regulates the mTOR pathway, mTOR signaling in AD astrocytes could be impaired, leading to reduction in amino acids pools. Leucine metabolism appears to be more affected in AD astrocytes than in AD neurons. It can be speculated that the metabolic alterations in astrocytes may be an early event in AD progression, possibly influencing neuronal metabolic and neurotransmitter homeostasis. The findings demonstrate reduced amino acid synthesis derived from leucine metabolism in AD astrocytes, possibly contributing further to neurotransmitter imbalances involved in AD.

Together, the results demonstrate extensive branched-chain amino acid metabolism in human astrocytes, identify disturbances in their metabolism in an AD pathological context, and highlight the need for further investigation on branched-chain amino acids metabolism, particularly in astrocytes, to possibly unravel additional pathological mechanisms implicated in neurodegenerative diseases.

## Data Availability Statement

The raw data supporting the conclusions of this article will be made available by the authors, without undue reservation.

## Ethics Statement

The studies involving human participants were reviewed and approved by the local Ethics Committee in Copenhagen (H-2-2011-104). The patients/participants provided their written informed consent to participate in this study. The animal study was reviewed and approved by the Danish National Ethics Committee and performed according to the European Convention (ETS 123 of 1986).

## Author Contributions

BA, CS, and JA: study concept and design. LP provided the brain tissue from patients. CS, KV, and JA: acquisition of data. CS, BA, KV, and JA: analysis and interpretation of data and drafting of the manuscript. BA, KF, HW, JA, and LP: critical revision of the manuscript for intellectual content. CS: statistical analysis. BA, JA, HW, and KF: obtained funding. BA, KF, and HW: study supervision. All authors contributed to the article and approved the submitted version.

## Funding

This study was supported by grants from The National Council of Science and Technology (CONACYT), (PhD Grant No: 2018-000009-01EXTF-00121 to CS), The Scholarship of Peter & Emma Thomsen (to JA), Aase and Ejnar Danielsen Foundation (Grant No: 10-002028 to JA), the Augustinus Foundation (Grant No: 17-4115 to JA), and Innovation Fund Denmark Brainstem (Grant No: 4108-00008B) and NeuroStem (to KF).

## Conflict of Interest

The authors declare that the research was conducted in the absence of any commercial or financial relationships that could be construed as a potential conflict of interest.

## Publisher's Note

All claims expressed in this article are solely those of the authors and do not necessarily represent those of their affiliated organizations, or those of the publisher, the editors and the reviewers. Any product that may be evaluated in this article, or claim that may be made by its manufacturer, is not guaranteed or endorsed by the publisher.
