# Supplementary material for: Functional Metabolic Mapping Reveals Highly Active Branched-Chain Amino Acid Metabolism in Human Astrocytes, Which Is Impaired in iPSC-Derived Astrocytes in Alzheimer's Disease
Source: Front Aging Neurosci. 2021 Sep 17;13:736580. doi: 10.3389/fnagi.2021.736580 (PMC8484639; doi:10.3389/fnagi.2021.736580)
Supplement: Supplementary file 1 [file Data_Sheet_1.docx]

**Supplementary material**

| Human and mouse cerebral cortical slices | | | | |
| --- | --- | --- | --- | --- |
| Amino acid  (nmol/mg) | Tissue origin | **[U-^13^C]leucine** | **[U-^13^C]isoleucine** | **[U-^13^C]valine** |
|  | Human | 32.48 ± 3.02 | 38.90 ± 9.84 | 39.77 ± 12.3 |
| *Aspartate* | Mouse | **50.05 ± 11.4*** | **66.37 ± 7.35*** | **69.94 ± 14.4*** |
|  | Human | 219.8 ± 37.5 | 274.4 ± 56.1 | 281.6 ± 61.5 |
| *Glutamate* | Mouse | 166.1 ± 20.3 | **213.1 ± 14.8*** | **191.7 ± 23.9*** |
|  | Human | 14.22 ± 4.73 | 14.40 ± 5.06 | 16.81 ± 7.40 |
| *Glutamine* | Mouse | 12.57 ± 2.64 | 11.06 ± 1.12 | 11.51 ± 2.23 |
|  | Human | 15.18 ± 3.45 | 22.74 ± 8.66 | 22.45 ± 6.61 |
| *GABA* | Mouse | 11.93 ± 1.25 | 16.40 ± 1.09 | **14.61 ± 1.57*** |
|  | Human | 79.06 ± 9.59 | 82.02 ± 15.8 | 136.8 ± 28.5 |
| *BCAA* | Mouse | **155.0 ± 26.6*** | **154.5 ± 37.4*** | **171.5 ± 23.3*** |

**Table. S1** Total intracellular amounts (nmol/mg protein) of amino acids after incubation with [U-^13^C]leucine, [U ^13^C]isoleucine and [U-^13^C]valine in human and mouse cerebral cortical slices. Values represent mean (±) SEM (n=6), *p<0.05, when compared with control (human cortical slices), analyzed by Student´s paired t-test.

| hiPSC-derived astrocytes | | | |
| --- | --- | --- | --- |
| Metabolite  labeling (%) | Group | **[U-^13^C]leucine** | **[U-^13^C]isoleucine** |
|  | WT | 4.36 ± 0.76 | 4.48 ± 0.63 |
| *α-ketoglutarate* | APP | 5.16 ± 0.86 | 4.33 ± 0.34 |
|  | PSEN-1 | 6.63 ± 0.64 | 5.17 ± 1.22 |
|  | WT | 2.38 ± 0.52 | 4.18 ± 1.06 |
| *Succinate* | APP | 1.43 ± 0.70 | 2.69 ± 0.99 |
|  | PSEN-1 | 2.0 ± 0.35 | 2.17 ± 0.61 |
|  | WT | 1.07 ± 0.08 | 1.12 ± 0.13 |
| *Fumarate* | APP | 1.14 ± 0.44 | 1.13 ± 0.52 |
|  | PSEN-1 | 1.51 ± 0.58 | 0.82 ± 0.62 |

**Table. S2** ^13^C metabolic enrichment (M+2) after incubation with [U-^13^C]leucine and [U-^13^C]isoleucine in hiPSC-derived astrocytes. Values represent mean (±) SEM (n=3), *p<0.05, when compared with control, analyzed by two-way ANOVA. No significant differences were found. *WT, wild type control; APP, amyloid precursor protein mutation; PSEN-1, presenilin 1 mutation.*

| hiPSC-derived astrocytes | | | |
| --- | --- | --- | --- |
| Metabolite  labeling (%) | Group | **[U-^13^C]valine** | **[U-^13^C]isoleucine** |
|  | WT | 1.05 ± 0.43 | 2.13 ± 0.40 |
| *α-ketoglutarate* | APP | 0.35 ± 0.20 | 1.45 ± 0.21 |
|  | PSEN-1 | 0.73 ± 0.26 | 1.71 ± 0.13 |
|  | WT | 0.48 ± 0.20 | 1.77 ± 1.16 |
| *Succinate* | APP | 0.46 ± 0.10 | 1.09 ± 0.10 |
|  | PSEN-1 | 0.45 ± 0.02 | 1.28 ± 0.09 |
|  | WT | 0.46 ± 0.02 | 1.99 ± 0.19 |
| *Fumarate* | APP | 0.72 ± 0.03 | 2.41 ± 0.27 |
|  | PSEN-1 | 0.76 ± 0.08 | 1.91 ± 0.52 |

**Table. S3** ^13^C metabolic enrichment (M+3) after incubation with [U-^13^C]valine and [U-^13^C]isoleucine in hiPSC-derived astrocytes. Values represent mean (±) SEM (n=3), *p<0.05, when compared with control, analyzed by two-way ANOVA. No significant differences were found. *WT, wild type control; APP, amyloid precursor protein mutation; PSEN-1, presenilin 1 mutation.*

| hiPSC-derived neurons | | | |
| --- | --- | --- | --- |
| Metabolite  labeling (%) | Group | **[U-^13^C]leucine** | **[U-^13^C]isoleucine** |
|  | WT | 6.34 ± 1.07 | 7.42 ± 0.68 |
| *Citrate* | APP | 6.73 ± 0.30 | 8.58 ± 0.91 |
|  | PSEN-1 | 10.7 ± 1.11 | 4.65 ± 1.67 |
|  | WT | 3.66 ± 0.23 | 5.07 ± 0.27 |
| *α-ketoglutarate* | APP | 3.95 ± 0.05 | 7.14 ± 0.16 |
|  | PSEN-1 | 3.47 ± 0.99 | 1.25 ± 0.91 |
|  | WT | 1.55 ± 0.13 | 2.42 ± 0.16 |
| *Succinate* | APP | 2.28 ± 0.15 | 1.66 ± 0.24 |
|  | PSEN-1 | 1.62 ± 0.69 | 1.03 ± 0.10 |
|  | WT | 0.00 ± 0.00 | 0.00 ± 0.00 |
| *Fumarate* | APP | **1.62 ± 0.12*** | **1.60 ± 0.14*** |
|  | PSEN-1 | 1.09 ± 0.77 | 0.63 ± 0.25 |
|  | WT | 1.87 ± 0.33 | 3.11 ± 0.15 |
| *Malate* | APP | 3.59 ± 1.47 | 2.76 ± 0.54 |
|  | PSEN-1 | 1.45 ± 0.53 | 2.05 ± 0.13 |

**Table. S4** ^13^C metabolic enrichment (M+2) after incubation with [U-^13^C]leucine and [U-^13^C]isoleucine in hiPSC-derived neurons. Values represent mean (±) SEM (n=3), *p<0.05, when compared with control, analyzed by two-way ANOVA. Increased fumarate labeling was found in APP neurons after incubation with [U-^13^C]leucine and [U-^13^C]isoleucine. *WT, wild type control; APP, amyloid precursor protein mutation; PSEN-1, presenilin 1 mutation.*

| hiPSC-derived neurons | | | |
| --- | --- | --- | --- |
| Metabolite  labeling (%) | Group | **[U-^13^C]valine** | **[U-^13^C]isoleucine** |
|  | WT | 3.74 ± 0.40 | 3.46 ± 0.46 |
| *Citrate* | APP | 2.60 ± 0.83 | 4.30 ± 0.32 |
|  | PSEN-1 | 0.97 ± 0.64 | 3.47 ± 1.45 |
|  | WT | 3.45 ± 0.13 | 2.35 ± 0.18 |
| *α-ketoglutarate* | APP | 1.93 ± 1.28 | 2.84 ± 0.30 |
|  | PSEN-1 | 1.42 ± 0.70 | 1.31 ± 0.65 |
|  | WT | 1.14 ± 0.08 | 1.74 ± 0.09 |
| *Succinate* | APP | 0.80 ± 0.25 | 1.11 ± 0.30 |
|  | PSEN-1 | 1.03 ± 0.22 | 2.34 ± 0.11 |
|  | WT | 0.12 ± 0.03 | 1.15 ± 0.10 |
| *Fumarate* | APP | 1.03 ± 0.22 | 1.40 ± 0.12 |
|  | PSEN-1 | 0.70 ± 0.18 | 1.29 ± 0.07 |
|  | WT | 1.15 ± 0.04 | 4.61 ± 0.11 |
| *Malate* | APP | 1.22 ± 0.40 | 4.16 ± 0.44 |
|  | PSEN-1 | 1.49 ± 0.22 | 5.70 ± 1.16 |

**Table. S5** ^13^C metabolic enrichment (M+3) after incubation with [U-^13^C]valine and [U-^13^C]isoleucine in hiPSC-derived neurons. Values represent mean (±) SEM (n=3), *p<0.05, when compared with control, analyzed by two-way ANOVA. No significant differences were found. *WT, wild type control; APP, amyloid precursor protein mutation; PSEN-1, presenilin 1 mutation.*

|  | Human and mouse cortical slices | | hiPSC- derived  astrocytes | | hiPSC- derived  neurons | |
| --- | --- | --- | --- | --- | --- | --- |
| Metabolite  labeling (%) | Group | **[U-^13^C]leucine** | Group | **[U-^13^C]leucine** | Group | **[U-^13^C]leucine** |
|  | Human | 4.35 ± 0.29 | WT | 2.78 ± 0.40 | WT | 2.10 ± 0.50 |
| *Citrate* | Mouse | **9.08 ± 0.48^#^** | APP | **4.80 ± 0.24*** | APP | 4.83 ± 2.17 |
|  |  |  | PSEN-1 | **4.87 ± 0.14*** | PSEN-1 | 8.88 ± 3.96 |
|  | Human | 1.79 ± 0.15 | WT | 2.48 ± 0.42 | WT | 3.60 ± 0.89 |
| *α-ketoglutarate* | Mouse | 2.75 ± 0.66 | APP | 1.89 ± 1.00 | APP | 5.24 ± 0.87 |
|  |  |  | PSEN-1 | 4.13 ± 1.21 | PSEN-1 | 2.51 ± 1.13 |
|  | Human | 0.23 ± 0.04 | WT | 1.53 ± 0.19 | WT | 0.58 ± 0.35 |
| *Succinate* | Mouse | 0.00 ± 0.00 | APP | **0.03 ± 0.03*** | APP | 0.61 ± 0.10 |
|  |  |  | PSEN-1 | 0.39 ± 0.21 | PSEN-1 | 0.30 ± 0.15 |
|  |  |  | WT | 0.15 ± 0.09 | WT | 0.00 ± 0.00 |
| *Fumarate* |  | ND | APP | 0.96 ± 0.56 | APP | 0.29 ± 0.29 |
|  |  |  | PSEN-1 | 0.88 ± 0.51 | PSEN-1 | 0.08 ± 0.08 |
|  | Human | 2.93 ± 0.46 | WT | 2.51 ± 0.22 | WT | 0.34 ± 0.27 |
| *Malate* | Mouse | **5.69 ± 0.66^#^** | APP | **1.55 ± 0.25*** | APP | 1.19 ± 0.46 |
|  |  |  | PSEN-1 | 1.94 ± 0.16 | PSEN-1 | 1.43 ± 1.21 |
|  | Human | 2.04 ± 0.35 | WT | 2.23 ± 0.23 | WT | 0.00 ± 0.00 |
| *Aspartate* | Mouse | **6.40 ± 0.59^#^** | APP | **0.86 ± 0.08*** | APP | 0.18 ± 0.18 |
|  |  |  | PSEN-1 | 1.47 ± 0.18 | PSEN-1 | 1.39 ± 1.39 |
|  | Human | 4.40 ± 0.24 | WT | 4.91 ± 0.16 | WT | 0.00 ± 0.00 |
| *Glutamate* | Mouse | **7.95 ± 0.35^#^** | APP | 4.94 ± 0.27 | APP | 0.91 ± 0.91 |
|  |  |  | PSEN-1 | 5.75 ± 1.12 | PSEN-1 | 0.30 ± 0.30 |
|  | Human | 5.51 ± 0.44 |  |  | WT | 0.95 ± 0.33 |
| *GABA* | Mouse | **13.78 ± 0.51^#^** |  | ND | APP | 0.43 ± 0.02 |
|  |  |  |  |  | PSEN-1 | 1.44 ± 0.98 |
|  | Human | 5.24 ± 0.56 | WT | 2.04 ± 0.11 |  |  |
| *Glutamine* | Mouse | **10.63 ± 0.48^#^** | APP | 0.22 ± 0.22 |  | ND |
|  |  |  | PSEN-1 | 0.50 ± 0.26 |  |  |

**Table. S6** ^13^C metabolic enrichment (M+1) after incubation with [U-^13^C]leucine in human and mouse cerebral cortical slices, hiPSC-derived astrocytes and hiPSC-derived neurons. Values represent mean (±) SEM (n=6), ^#^p<0.0001 when compared with control (human cortical slices), and (n=3), *p<0.05 when compared with control (wild type), analyzed by two-way ANOVA. No significant differences were found in hiPSC-derived neurons.
